# Patient–Nurse Ratio is Related to Nurses’ Intention to Leave Their Job through Mediating Factors of Burnout and Job Dissatisfaction

**DOI:** 10.3390/ijerph16234801

**Published:** 2019-11-29

**Authors:** Yi-Chuan Chen, Yue-Liang Leon Guo, Wei-Shan Chin, Nai-Yun Cheng, Jiune-Jye Ho, Judith Shu-Chu Shiao

**Affiliations:** 1 School of Nursing, College of Medicine, National Taiwan University (NTU), No.1, Ren-Ai Rd. Sec. 1, Taipei 10051, Taiwan; d05426004@ntu.edu.tw; 2 Department of Environmental and Occupational Medicine, College of Medicine, National Taiwan University (NTU), No.1, Ren-Ai Rd. Sec. 1, Taipei 10051, Taiwan; leonguo@ntu.edu.tw; 3 Department of Environmental and Occupational Medicine, National Taiwan University Hospital (NTUH), No.7, Chung-Shan South Rd., Taipei 10002, Taiwan; 4 School of Nursing, College of Nursing, Taipei Medical University (TMU), No. 250, Wuxing St., Taipei 11031, Taiwan; weishanchin@tmu.edu.tw; 5 Institute of Labor, Occupational Safety and Health (ILOSH), Ministry of Labor, No. 99, Lane 407, Hengke Rd., New Taipei City 22143, Taiwan; acer3192@mail.ilosh.gov.tw (N.-Y.C.); hjj@mail.ilosh.gov.tw (J.-J.H.); 6 Department of Nursing, National Taiwan University Hospital (NTUH), No.7, Chung-Shan South Rd., Taipei 10002, Taiwan; 7 Occupational Health Nursing and Education Association of Taiwan (OHNEAT), No.1, Ren-Ai Rd. Sec. 1, Taipei 10051, Taiwan

**Keywords:** burnout, intention to leave, job dissatisfaction, nurse, patient–nurse ratio

## Abstract

In healthcare settings, nurses’ workload, burnout, and job satisfaction are associated to the patient–nurse ratio. Whether this ratio also affects their intention to leave the nursing profession, along with the underlying stress pathway, remains unclear. This study aimed to investigate the effects of the patient–nurse ratio on nurses’ intention to leave and considering the mediating roles of burnout and job dissatisfaction. The study analyzed the data of two pooled cross-sectional surveys collected in 2013 and 2014. Measures were obtained by a structure questionnaire, which queried the average daily patient–nurse ratio (ADPNR), nurses’ personal burnout, client-related burnout, job dissatisfaction, intention to leave, and other demographics. ADPNRs were standardized according to hospital levels. Multiple regression models examined mediation hypotheses, and a percentile bootstrap confidence interval was applied to determine the significance of indirect effects. A total of 1409 full-time registered nurses in medical and surgical wards of 24 secondary or tertiary hospitals in Taiwan completed self-administered questionnaires. Most of the participants were female (97.2%), and the mean age was 29.9 years. The association between the standardized ADPNR and intention to leave their job was significantly mediated by personal burnout, client-related burnout, and job dissatisfaction. Higher standardized ADPNRs predicted higher levels of personal burnout, client-related burnout, and job dissatisfaction, each of which resulted in higher levels of intention to leave the current job. The results highlight that appropriate patient–nurse ratio standards may be further discussed by selecting personal burnout, client-related burnout, and job dissatisfaction as indicators.

## 1. Introduction

Given that direct labor costs are the major budget items, hospitals face the dilemma between reducing or limiting direct labor costs and providing better care quality [1]. Although some hospitals reduce nurse staffing to minimize cost, nurse staffing has been reported to be influential not only in hospitalized patients’ prognoses and safety [2,3,4] but also in hospitals’ financial performance [5]. Furthermore, studies show that hospitals with better nurse staffing can alleviate nurses’ workload [1,6], attract nurses to practice [7], and diminish workplace injury and illness rates [8]. When experiencing the predicament of a nursing shortage, following the lead of the state of Victoria in Australia, many governments have made legislations for nurse staffing [9,10,11,12]. Some governments use a direct patient–nurse ratio (PNR) to represent the total number of patients taken care of by each nurse in each shift [11,13]. Japan offers a fee schedule for hospitals with different levels of nurse staffing: More sufficient staffing, more reimbursement [10]. In Taiwan, the standards, average daily PNRs (ADPNRs), are set in accordance with hospital levels, and have been effective since 1 May 2019 for medical and surgical wards. The ADPNR is calculated by the total number of beds*bed occupancy rate per month*3 (shifts)/average total number of daily nurses per month; head nurses, nurse practitioners, and student nurses are excluded. The ADPNRs of tertiary hospitals, secondary hospitals, and primary hospitals should be less than or equal to 9, 12, and 15, respectively [14].

On the International Workforce Forum of International Council of Nurses (ICN), nursing leaders have appealed to governments to take action to ensure safe staffing levels and decent working conditions for patients and nurses. As the chief executive officer of ICN accentuated, nurses are not only essential for providing safe and high-quality care but also for teaching the next generations. Besides, we risk losing capable nurses faster than the juniors are trained [15]. A surveyed conducted among 10 European countries showed that nurses’ intention to leave the nursing profession ranged from 5% to 17% [16]. In Taiwan, the average resignation rate ranged from 9.12% to 12.00% in 2013 to 2016 [17]. Apparently, nurses’ resignation is a crucial issue, and researchers have attempted to identify its predictors and prevention strategies. Factors regarding nurses’ intention to leave may be grouped into two dimensions: (1) Demographics, such as age, gender, marital status, educational level, and work tenure [18,19]; and (2) hospital context, such as inadequate nurse staffing, workload, workplace injustice, workplace violence, interpersonal relationship, burnout, and job dissatisfaction [16,19,20,21,22]. Being married [18], satisfactory workplace justice, nurse–physician relationships, leadership, and participation in hospital affairs [16,19,21] were found to correlate negatively with intention to leave. By contrast, less job control, higher psychological demand, perceived burnout, and low job satisfaction correlated positively with intention to leave [19,20,21]. On the other hand, the specific gender [16,18], age group, and educational level [18,19] that had a higher intention to leave was inconsistent in previous studies. Besides, researchers have suggested that adequate nurse staffing is negatively associated with nurses’ burnout and job dissatisfaction [20,22], which could be predictors of nurses’ intention to leave their present jobs within one year [2]. Barrientos-Trigo et al. reviewed kinds of interventions to improve nursing working conditions, indicating that macro-management level interventions, such as having better PNR, would improve staff outcomes [23]. The PNR was found to be associated with nurses’ workload [24,25], and was used as an indicator to inspect nurses’ perception and appraisal of their jobs [22]. Using meta-analysis, Shin et al. indicated that the PNR was correlated to nurses’ level of burnout, job dissatisfaction, and intention to leave [22]. Additionally, the effects of the work environment and PNR on nurses’ burnout, job dissatisfaction, and intention to leave could not be diminished even when considered with wage simultaneously [26].

Burnout is a state of physical, emotional, and mental exhaustion and fatigue, which usually derives from work-related demands in a person’s life [27]. Recognized as a result of chronic workplace stress without successful management, burnout will be a medical diagnosis in the International Classification of Diseases 11th Revision (ICD-11) from 1 January 2022 [28]. Thus, the PNR is plausibly a psychosocial hazard of the nursing practice environment, which may lead to organizational personnel loss, and consequently, affect patient care quality. To our knowledge, no valid instrument to use when estimating nurses’ leaving tendency before real resignation currently exists. We focus on the macro level of improving the nursing practice environment as the classification of Barrientos-Trigo et al. [23] proposed and agree with the viewpoint of Aiken et al. [2]. Hence, burnout and job dissatisfaction were chosen to be our mediators while the relationship between PNR and intention to leave was estimated.

In the safe nurse staffing ratio campaign, the California Nurses Association proposed and strove for ratios that referred to nurses’ experience [29], and finally made the California Ratio Law effective. Then, studies focused on the mandatory ratios in California were reported [30]. However, many previous studies used a cross-sectional design or secondary data with logistic regression analysis, which limited the causal inference of PNRs to patients’ outcomes or nurses’ occupational-related psychosocial status and intention to leave. We believe that the effectiveness of the PNR legislation needs to be assessed for further amendment, and for governments or facilities to set their own PNR standards as a reference.

**Figure 1:**
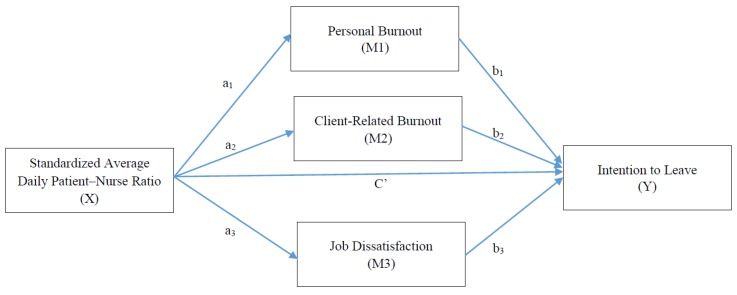
The mediation model of the relationship between the standardized average daily patient–nurse ratio and intention to leave through personal burnout, client-related burnout, and job dissatisfaction.

To realize the dose–response relationship between the PNR and its influences, we chose the PNR as the antecedent, and used nurses’ perceived burnout, job dissatisfaction, and intention to leave as consequents in a mediated model for two major reasons. First, the data collection was relatively accessible and less time and money consuming than a cohort study. Although we also used a cross-sectional design, the mediated model, without logical controversy, is eligible for an examination of the causal mechanism [31]. That is, we believe that massive PNR may lead to nurses’ burnout, job dissatisfaction, and intention to leave their jobs, but the perceived burnout, job dissatisfaction, and intention to leave of nurses cannot reversely contribute to the PNR. Second, California’s ratios were determined according to nurses’ work experience before the publication of rigorous study results [29]. Factors in the workplace associated with nurses’ resignation are complex, especially those factors related to personnel’s interactions and patients’ severity. Nevertheless, we believe an adequate PNR is a comparatively precise goal to strive for better working conditions. Considering Taiwan has the legislation of ADPNR, we aimed to examine the relationship between ADPNR and nurses’ intention to leave, as well as considering the mediating role of burnout and job dissatisfaction. A model of the examined pathways to intention to leave is illustrated in Figure 1. Our hypotheses were as follows:

**Hypothesis** **1.** *Personal burnout positively mediates the relationship between ADPNR and intention to leave*.

**Hypothesis** **2.** *Client-related burnout positively mediates the relationship between ADPNR and intention to leave*.

**Hypothesis** **3.** *Job dissatisfaction positively mediates the relationship between ADPNR and intention to leave*.

**Hypothesis** **4.** *ADPNR is positively and directly related to intention to leave, as well as through mediating factors of personal burnout, client-related burnout, and job dissatisfaction indirectly*.

## 2. Materials and Methods

### 2.1. Participants and Data Collection

Two cross-sectional investigations were conducted in 2013 and 2014 with a structured and self-administered questionnaire after the Research Ethics Committee (REC) of National Taiwan University Hospital approved the protocols (protocol numbers: 20130807RINC and 201407075RINA). Electable hospitals, accredited by the Joint Commission of Taiwan and no overlap between these two years, were stratified by random sampled according to region (north, central, south, and east) and in proportion by hospital level. In 2013, the number of nurses practicing in primary, secondary, and tertiary hospitals was 21.6%, 44.0%, and 34.4%, respectively. Managers of the sampled hospitals were invited to approve the study via a phone call, and questionnaires were mailed to the participating hospitals according to the proportions of hospital levels. Then, questionnaires were delivered to nurses by coordinators. To analyze the effects of ADPNRs, this study focused on full-time registered nurses who worked in medical or surgical wards because ADPNR standards are presently only in those units in Taiwan. Although nurses of primary, secondary, and tertiary hospitals initially acceded to the investigation, patients prefer to have medical care in tertiary and secondary hospitals instead of primary hospitals because national health insurance makes it affordable. Accordingly, the bed occupancy rates and PNRs of primary hospitals may vary largely among institutions. Exemption of written consent was approved by the REC. The returned questionnaires were regarded as nurses’ willingness to participate in the study.

### 2.2. Measures

#### 2.2.1. ADPNR

In this study, nurses were asked to answer a question, “Generally speaking, how many patients do you take care of in day shift, evening shift and night shift, respectively?”. The equation used to calculate ADPNR can be specified as follows:

ADPNR=1(1D+1E+1N)3,

where D, E, and N are the numbers of patients cared for by each nurse in the day shift, evening shift, and night shift, respectively. Reciprocals of the denominator represent the attention each patient received from the responsible nurse in each shift. Then, the average of the sum of the reciprocal in each shift was used to describe the average daily nurses’ care of patients. Last, the inverse of the denominator stands for the ADPNR. For example, if a participant answered that he/she usually took care of 6, 12, and 18 patients in a day, evening and night shift, respectively, then the ADPNR=1(16+112+118)3=9.8.

Referring to the study of McHugh and Ma [26], nursing staffing was analyzed after standardization, and we believe this transformation made staffing more comparable among different hospital levels. As for standardized ADPNR, if the ADPNR of a participant who worked in a tertiary hospital was 9.8, then the standardized ADPNR was calculated as 9.8/9 = 1.09, which meant the participant usually experienced 1.09 times of ADPNR then the standard patient–nurse ratio in Taiwan. The test–retest reliability of ADPNR, measured by Pearson’s correlation coefficient, among 48 nurses measured within two weeks was 0.92.

#### 2.2.2. Intention to Leave

Intention to leave was measured by a rating and the items included the frequency of quitting the job [32]. Thus, we created the following two items to assess the intention to leave the current nursing job: (1) “Please rate your intention to leave on a scale of 0 to 10, with 0 being no intention to leave and 10 being highly considering leaving”; and (2) “How often do you think about leaving your job?”. Answers of “never”, “every month”, “every week”, and “every day” were coded from 1 to 4. Then, multiplied scores of those two items ranged from 0 to 40, as the level of intention to leave. The test-retest reliability (Pearson’s correlation coefficients) of the two items and the multiplied score in our study were 0.76, 0.69, and 0.74, respectively. To be comparable, the multiplied score of intention to leave was normalized on a scale from 0 to 100.

#### 2.2.3. Mediators

Personal burnout and client-related burnout are two of the four subscales in the Chinese version of the Copenhagen Burnout Inventory (C-CBI) [33]. As defined by Kristensen et al. [27], personal burnout is “the degree of physical and psychological fatigue and exhaustion experienced by the person”, while client-related burnout is “the degree of physical and psychological fatigue and exhaustion that is perceived by the person as related to his/her work with clients”. Containing five and six items, respectively, personal burnout and client-related burnout subscales were used to assess the frequencies of particular events within the preceding week on a 5-point Likert scale (“never” to “always” were scored as 0 to 4). Both the total score of personal burnout and client-related burnout ranging from 0 to 100 refer to the scoring method of Yeh et al. [33]. The five items measuring personal burnout included “how often do you feel tired”; “how often are you physically exhausted”; “how often are you emotionally exhausted”; “how often do you think: ‘I can’t take it anymore’”; and “how often do you feel weak and susceptible to illness”. The six items of the client-related burnout subscale were “do you find it hard to work with clients”; “does it drain your energy to work with clients”; “do you find it frustrating to work with clients”; “do you feel that you give more than you get back when you work with clients”; “are you tired of working with clients”; and “do you sometimes wonder how long you will be able to continue working with clients” [27,33]. The term “client” could be patient, student, etc. [27]. In the process of developing C-CBI, Yeh et al. [33] used work-related factors and individuals’ perceptions to show the concurrent criterion validity. Personal burnout and client-related burnout were found strongly and positively correlated with psychological distress, and moderately and positively correlated with perceived work stress and job demand, but negatively correlated with perceived health status. The internal consistency (Cronbach’s α) coefficients of personal burnout and client-related burnout subscales ranged from 0.90 to 0.92 in male and female participants [33].

Job dissatisfaction was valued by the item of “Generally speaking, are you satisfied with your job?” Answers of “very satisfied”, “somewhat satisfied”, “average”, “somewhat unsatisfied”, and “very unsatisfied” to the question were coded from 1 to 5. The Pearson’s correlation coefficient for the test–retest reliability in our pilot study was 0.82. To be comparable among the consequent factors of ADPNR, the score of job dissatisfaction was normalized on a scale from 0 to 100.

#### 2.2.4. Other Covariates. 

As mentioned previously, demographics and hospital context, such as age, working tenure, educational level in nursing, marital status, sleeping hours, and working hours, were indicated to be associated with nurses’ burnout, job satisfaction, and intention to stay or leave the job. Additionally, considering the ownership of hospitals might affect the leadership and nurses’ decision-making in public affairs, the ownership of hospitals was included as a covariate. All of these variables were collected in the questionnaire. Bivariate analyses and univariate regressions were conducted to determine which covariates could be retained in the mediation model. Two variables, current working tenure and total working tenure, were excluded from the model for multicollinearity by using Pearson correlation coefficients and the variance inflation factor [34]. The following covariates were included for modeling and were coded with a dummy: Hospital ownership (public, private), age (≤25, 26–35, ≥36), major shift in past three months (day, evening, night, rotating), education achievement (junior college, college, or above), marital status (single, married), sleeping hours per working day (<6, ≥6), and weekly working hours (<55, ≥55). The effects of age and weekly working hours on personal burnout, client-related burnout, job dissatisfaction, and intention to leave determined cut-off points for categorizations by using the points of inflection as a reference. The cut-off point of sleeping hours per working day was set by referring to the study of Chin et al. [35].

The research team members conducted a pretest of the questionnaire as well as expert panel discussions for suggestions. The content validity index of the questionnaire, assessed by three professionals in the fields of nursing, psychiatry, and occupational medicine, was 0.89 after revisions of the expert panel discussions.

### 2.3. Analytical Strategy

Descriptive statistics and correlations were used to describe the means, standard deviations, and associations among target variables. To verify the hypothesized mediational associations, mediation analyses were implemented using IBM SPSS statistics 25 software (SPSS, Inc., Chicago, IL, USA) and PROCESS macro v3.3 [36,37]. Figure 1 illustrates the proposed pathways from standardized ADPNR to intention to leave. Four regression models were established and examined. First, the association between standardized ADPNR and personal burnout (a_1_ path), client-related burnout (a_2_ path), and job dissatisfaction (a_3_ path) were estimated, respectively. Second, the associations of nurses’ intention to leave with standardized ADPNR (c’ path, also known as the direct effect), personal burnout (b_1_ path), client-related burnout (b_2_ path), and job dissatisfaction (b_3_ path) were estimated. All models adjusted for hospital ownership, age, major shift in past three months, education achievement, marital status, sleeping hours per working day, and weekly working hours. The indirect effects of the three mediators were estimated as the product of the regression coefficients linking standardized ADPNR to intention to leave through personal burnout (a_1_b_1_), client-related burnout (a_2_b_2_), and job dissatisfaction (a_3_b_3_). The completely standardized effect size was estimated by 95% confidence intervals (CIs) with a bootstrap of 5000 times [36]. A significant indirect effect was presumed when the 95% CIs did not contain 0.

## 3. Results

A total of 680 questionnaires were disseminated to 54 primary hospitals and 399 were completed satisfactorily. The ADPNRs of primary hospitals ranged from 4.6 to 25.7 (mean 12.9, SD 3.7). Thus, data of primary hospitals were excluded from the final analysis. Overall, in tertiary and secondary hospitals, 1004 questionnaires were disseminated and 844 were returned at the end of 2013; 1378 nurses were then recruited, of whom 1190 returned the questionnaires in 2014. After the exclusion of nurse managers, nurse practitioners, units out of medical or surgical wards, shifts out of 8 h, and incomplete questionnaires, a total of 1409 questionnaires from 7 tertiary and 17 secondary hospitals were deemed eligible for the analysis. The total effective response rate was 59.2%.

### 3.1. Descriptive Analyses

The participants’ demographics are shown in Table 1. The majority of the study population were female (*n* = 1369, 97.2%), and the mean age was 29.9 years old. Most of them were single (*n* = 1006, 71.4%), had a college or above educational achievement (*n* = 1003, 71.2%), and had an average current and total work tenure of 6.8 and 7.9 years, respectively. The participants practiced in secondary hospitals (51.3%) and tertiary hospitals (48.7%) almost equally. During the questionnaire survey, 33.6% of the participants reported that they had a rotating shift as the major shift in the past three months. The ADPNRs of secondary and tertiary hospitals were 13.1 and 10.7, respectively. Of the participants from secondary and tertiary hospitals, the average standardized ADPNRs were 1.1 (73.7% > 1.0) and 1.2 (97.4% > 1.0), respectively. In addition, the mean number of working hours per day was 9.7; consequently, the mean number of weekly working hours was 49.6. The nurses slept 6.9 h averagely. For personal burnout, client-related burnout, job dissatisfaction, and intention to leave, all with a total score range of 0 to 100, the participants got 64.3, 49.5, 52.3, and 37.3, respectively

Table 2 shows the odds ratio of potential confounders to our interested variables. Referring to Chin et al. [35], cut-off values were set at the 90th percentile for personal burnout and client-related burnout, as well as intention to leave. Participants who answered, “somewhat unsatisfied” and “very unsatisfied” to their jobs were regarded as having job dissatisfaction. In model 1, univariate logistic regression was implemented; then significant predictors were put into multiple logistic regression. We ran adjusted odds ratios (AOR) of ADPNR and standardized ADPNR in model 2 and model 3, respectively. After adjusting for significant variables, both ADPNR and standardized ADPNR had effects on higher personal burnout and higher client-related burnout among nurses. Besides, when ADPNR was worse than the Taiwanese regulation—standardized ADPNR more than 1—nurses had a higher risk of personal burnout (AOR = 1.88, 95% confidence interval, CI = 1.10–3.44). For client-related burnout, a higher risk was also seen in a standardized ADPNR more than 1 (AOR = 1.78, 95% CI = 1.04–3.25). For job dissatisfaction, neither ADPNR nor standardized ADPNR had statistically significant effects. For intention to leave, nurses with higher ADPNR may have a higher risk of quitting (AOR = 1.14, 95% CI = 1.03–1.27). All of the potential confounders were retained for mediation analysis because they had significant predictions of our interested consequent variables.

### 3.2. Mediation Analyses

Table 3 presents the effects of the standardized ADPNR on nurses’ intention to leave via personal burnout, client-related burnout, and job dissatisfaction. All models were adjusted by the covariates mentioned above.

Mediation through personal burnout. After controlling the covariates, standardized ADPNR was significantly associated with personal burnout (estimate for a_1_ = 15.06, *p* < 0.001). Besides, personal burnout was positively associated with intention to leave (estimate for b_1_ = 0.33, *p* < 0.001). The results showed that the association between standardized ADPNR and intention to leave was significantly mediated by personal burnout as the 95% bootstrap CI of the indirect effects did not include 0 (estimate for a_1_b_1_ = 4.97, 95% bootstrap CI = 2.36–8.00). The completely standardized effect size of personal burnout on intention to leave was 0.03 (95% bootstrap CI = 0.01–0.05). Thus, hypothesis 1 was supported.

Mediation through client-related burnout. Likewise, standardized ADPNR was positively associated with client-related burnout (estimate for a_2_ = 13.15, *p* < 0.001), and higher client-related burnout was associated with a higher level of intention to leave (estimate for b_2_ = 0.11, *p* < 0.01). The significant 95% bootstrap CI for the indirect effect noted that the association between standardized ADPNR and intention to leave was mediated by client-related burnout (estimate for a_2_b_2_ = 1.45, 95% bootstrap CI = 0.32–2.95). The completely standardized effect size of client-related burnout on intention to leave was 0.01 (95% bootstrap CI = 0.00–0.02). Thus, hypothesis 2 was supported.

Mediation through job dissatisfaction. The mediating model suggested that standardized ADPNR was positively associated with job dissatisfaction (estimate for a_3_ = 12.45, *p* < 0.001). Furthermore, as the indirect effect was significant (estimate for b_3_ = 0.41, *p* < 0.001), nurses with higher job dissatisfaction were more likely to have higher levels of intention to leave. The indirect effect presented that the association between standardized ADPNR and intention to leave was mediated by job dissatisfaction (a_3_b_3_ = 5.10, 95% bootstrap CI = 1.63–8.70). The completely standardized effect size of personal burnout on intention to leave was 0.03 (95% bootstrap CI = 0.01–0.05). Hence, hypothesis 3 was supported.

Total effect of standardized ADPNR was the sum of the indirect effects plus the direct effect of standardized ADPNR, and the latter one was the estimate for the path of c’ = 4.77 (95% bootstrap CI = −2.80–12.35). Hence, the total effect of standardized ADPNR = a_1_b_1_ + a_2_b_2_ + a_3_b_3_ + c’ = 4.97 + 1.45 + 5.10 + 4.77 = 16.29 (95% bootstrap CI = 7.19–25.34). The completely standardized effect size of standardized ADPNR and the mediators was 0.09 (*p* < 0.001, data not shown). Although standardized ADPNR could affect nurses’ intention to leave through mediating factors of personal burnout, client-related burnout, and job dissatisfaction indirectly, the direct effect of standardized ADPNR (c’) was not statistically significant. Thus, hypothesis 4 was not fully supported.

Hypotheses 1, 2, and 3 were supported in the mediation model. In summary, the results indicated that personal burnout, client-related burnout, and job dissatisfaction significantly mediated the effect of standardized ADPNR on nurses’ intention to leave.

## 4. Discussion

Among Taiwanese nurses working at secondary and tertiary hospitals, we examined the effect of nurse staffing on intention to leave. Although the impacts of improved staffing on nursing quality and patient safety might be controversial [30], confirmation of a relationship between PNR and nurses’ intention to leave might be coherent. A qualitative study among 12 nurses in acute care inpatient settings found that understaffing and perceived high PNR may increase workload demands, which could further contribute to their decisions to leave [38]. A meta-analysis of 13 cross-sectional studies showed higher PNR had a higher risk for burnout, job dissatisfaction, and intent to leave, with significant effect sizes ranging from 1.05 to 1.08 [22]. One of our findings was similar to Dutra et al. [39], in which staffing adequacy could not predict nurses’ job dissatisfaction but could estimate intention to leave while adjusting for other personal and organizational factors. However, our study added information by implementing mediation analyses instead of logistic regression, suggesting that standardized ADPNR may affect nurses’ intention to leave through mediating effects of personal burnout, client-related burnout, and job dissatisfaction.

We proposed a novel equation to calculate ADPNR. Whereas the equation does not consider bed occupancy rate as the legislation in Taiwan, it may be more representative of common ADPNR, and approximate to nurse staffing legislations in other countries. For example, the multinational nurse forecasting in Europe (RN4CAST) study investigated participating nurses’ total patient and nurse numbers, respectively, to calculate the PNR on the last shift [40]. Similarly, in California, the legislated PNRs are also based on the maximum patient numbers that each nurse can be in charge of [13].

In our study, with a nationwide representative sampling, the overall ADPNR of medical and surgical wards was 11.9 (13.1 in secondary hospitals and 10.7 in tertiary hospitals), which was higher than the survey of Aiken et al. [40] in nine European countries, from 5.2 (Norway) to 10.8 (Belgium), but similar to Spain (12.7). Then, the ADPNR was standardized in accordance with Taiwanese regulation. Most of the participating secondary and tertiary hospitals did not meet the Taiwanese legislation of ADPNR.

Information of the coefficients of nurse staffing on burnout, job dissatisfaction, and intention to leave is deficient; however, our result is supported by studies regarding the effects of PNR on burnout and job dissatisfaction [2,22]. Although PNR was not statistically significantly associated with intention to leave, burnout was consistently associated with nurses’ intention to leave the profession among 10 European countries [16]. Applying a mediation analysis, we found support for hypotheses 1, 2, and 3: The standardized ADPNR affected nurses’ intention to leave their job positively, and the association was mediated by personal burnout, client-related burnout, and job dissatisfaction. In our study, the total effect of standardized ADPNR was 16.29—that is, regardless of other factors, two nurses who differ by one unit in standardized ADPNR were estimated to differ by 16.29 units in their reported intention to leave. On the other hand, Table 3 also presents the completely standardized effects of the mediators (i.e., personal burnout, client-related burnout, and job dissatisfaction) on intention to leave. Because the scores of all mediators were normalized from the range of 0 to 100, the estimated completely standardized effects could indicate the degree of indirect effects of these mediators: Personal burnout (0.03) and job dissatisfaction (0.03) were more influential than client-related burnout (0.01).

Although the effect of standardized ADPNR on intention to leave in our model was completely mediated by the mediators, the use of a different mediator may challenge the result [36]. The relatively small R-square in Table 3 implies the potential existence of other influential factors. Studies have indicated that workplace violence [21,41] and organizational support are positively associated with burnout and negatively associated with job satisfaction, and could be predictors of intention to leave [21]. Moreover, low job control, low workplace justice, and high psychological demand were predictors of having a higher intention of leaving the nursing profession [19]. Apart from PNR, the nursing work environment is also predominantly associated with burnout, job dissatisfaction, and intent to leave [26]. Further research will be helpful to clarify these and other possible mediators linking PNR to intent to leave among nurses for the development of PNR legislation.

Despite these contributions, our results should be interpreted with caution because of several limitations. First, the healthy worker effect or healthy worker survival effect [36] might constrain the generalization to all hospital nurses. Nurses who could not adapt to the practice environment may had left the profession, or transferred to better-conditioned workplaces, and were unavailable for this study. Second, the results showed that standardized ADPNR had an indirect effect on nurses’ intention to leave through personal burnout, client-related burnout, and job dissatisfaction. Although the use of a cross-sectional study for causal inference is considered inappropriate and mediation analysis is ultimately a causal explanation, we agree that it is our thoughts and interpretation that makes the mathematical procedures meaningful as Hayes claimed [42]. Furthermore, personal burnout, client-related burnout, and job dissatisfaction, to our knowledge, would not make it possible to affect ADPNR. Thus, we hypothesized ADPNR as the antecedent variable instead of a mediator in the model. Last, this study lacks some information, such as specific work unit, bed occupancy rates, and total numbers of beds and nurses in those units. Hence, the effects of PNR calculated by bed occupancy rates could not be examined in our study. Regardless of these limitations, our study has several strengths. The data for analysis in this article were part of our study that was implemented to survey nurses’ work environment. The items of PNR were arranged with demographics, such as age, gender, work tenure, and unit, in the last part of the questionnaire. Thus, participants might not connect PNR with items of personal burnout, client-related burnout, job dissatisfaction, and intention to leave. The potential impact of response bias may have hence been lessened. In addition, the effectiveness of legislated PNR may be examined through a nature experiment, e.g., before and after legislation implementation. In our study, we examined a theoretical causal model, which would help further researchers establish advanced models and provide suggestions to policy makers to determine appropriate PNRs by using staff’s outcomes as a convenient and convincing reference.

## 5. Conclusions

We found that an increased patient–nurse ratio would induce nurses’ intention to leave their job. This effect was mainly mediated by personal burnout, client-related burnout, and job dissatisfaction. We recommend that policymakers and researchers should determine an appropriate standard patient–nurse ratio with personal burnout, client-related burnout, and job dissatisfaction, and periodically assess nurses’ intention to leave with the patient–nurse ratio as well as these three mediators. Such an effort will potentially be helpful to establish or amend existing legislation regarding the patient–nurse ratio in the future.

## Figures and Tables

**Table 1:** Participants’ demographics.

Variable	All Participants (*N* = 1409)			
	*n*	%	Mean	SD
Gender				
Male	21	1.5		
Female	1369	97.2		
Missing	19	1.4		
Age (years)			29.9	6.9
≤25	480	34.1		
26–35	650	46.1		
≥36	267	18.9		
Missing	12	0.9		
Marital status				
Single	1006	71.4		
Married	401	28.5		
Missing	2	0.1		
Education achievement				
Under college	402	28.5		
College or above	1003	71.2		
Missing	4	0.3		
Current work tenure (years)			6.8	6.3
<5	740	52.5		
5–15	498	35.3		
>15	167	11.9		
Missing	4	0.3		
Total work tenure (years)			7.9	7.0
<5	669	47.5		
5–15	512	36.3		
>15	224	15.9		
Missing	4	0.3		
Hospital level				
Secondary	723	51.3		
Tertiary	686	48.7		
Unit				
Medical ward	801	56.9		
Surgical ward	608	43.2		
Major shift in past three months				
Day shift	425	30.2		
Evening shift	186	13.2		
Night shift	313	22.2		
Rotating shift	473	33.6		
Missing	12	0.9		
Average daily patient–nurse ratio (ADPNR)			11.9	1.9
Secondary hospital			13.1	2.0
Tertiary hospital			10.7	0.7
Standardized average daily patient–nurse ratio (standardized ADPNR)			1.1	0.1
Secondary hospital			1.1	0.2
Tertiary hospital			1.2	0.1
Working hours/day			9.7	1.2
Working hours/week			49.6	9.0
Sleeping hours/day			6.9	1.4
Personal burnout (range: 0–100)			64.3	20.5
Client-related burnout (range: 0–100)			49.5	19.3
Job dissatisfaction (range: 0–100)			52.3	19.0
Intention to leave (range: 0–100)			37.3	26.4

**Table 2:** Odds ratio of potential confounders with burnout, job dissatisfaction, and intention to leave.

Variable	Personal Burnout			Client-Related Burnout			Job Dissatisfaction			Intention to Leave		
Variable	Odds Ratio (95% CI)											
	M1	M2	M3	M1	M2	M3	M1	M2	M3	M1	M2	M3
**ADPNR**	1.19 (1.10–1.28)	1.13 (1.03–1.25)		1.15 (1.07–1.24)	1.17 (1.06–1.29)		1.10 (1.03–1.17)	1.08 (1.00–1.18)		1.15 (1.05–1.24)	1.14 (1.03–1.27)	
*p* Value	<0.001	0.011		<0.001	0.002		0.003	0.053		0.001	0.014	
**Standardized ADPNR**												
≤1	1.00		1.00	1.00		1.00	1.00		1.00	1.00		1.00
>1	1.83 (1.09–3.31)		1.88 (1.10–3.44)	1.77 (1.06–3.14)		1.78 (1.04–3.25)	1.27 (0.88–1.90)		1.30 (0.88–1.97)	1.71 (0.91–3.58)		1.76 (0.93–3.70)
*p* Value	0.022		0.020	0.027		0.036	0.210		0.186	0.097		0.084
**Ownership**												
public	1.00			1.00			1.00	1.00	1.00	1.00		
private	1.19 (0.86–1.64)			1.19 (0.87–1.63)			1.39 (1.07–1.79)	1.13 (0.85–1.51)	1.16 (0.87–1.53)	1.24 (0.85–1.80)		
*p* Value	0.281			0.281			0.012	0.406	0.311	0.257		
**Major shift in the past three months**												
Day shift	1.00	1.00	1.00	1.00	1.00	1.00	1.00			1.00		
Evening shift	0.42 (0.19–0.83)	0.44 (0.20–0.90)	0.44 (0.19–0.89)	0.55 (0.28–1.01	0.53 (0.25–1.05)	0.52 (0.25–1.03)	0.97 (0.62–1.49)			1.22 (0.63–2.27)		
Night shift	1.21 (0.77–1.89)	0.97 (0.60–1.58)	0.90 (0.56–1.47)	1.21 (0.78–1.87)	1.04 (0.64–1.69)	1.01 (0.62–1.64)	1.10 (0.77–1.58)			1.27 (0.75–2.14)		
Rotating shift	1.61 (1.10–2.39)	1.08 (0.71–1.64)	1.12 (0.74–1.71)	1.30 (0.89–1.92	0.93 (0.60–1.46)	0.98 (0.63–1.53)	1.35 (0.98–1.86)			1.17 (0.73–1.88)		
*p* Value	<0.001	0.076	0.047	0.024	0.254	0.228	0.217			0.817		
**Age**												
≤25	1.00			1.00	1.00	1.00	1.00			1.00		
26–35	1.38 (0.96–2.00)			1.42 (1.01–2.02)	1.45 (0.99–2.14)	1.45 (0.99–2.13)	0.97 (0.73–1.28)			1.21 (0.81–1.82)		
≥36	1.11 (0.68–1.77)			0.47 (0.26–0.82)	0.44 (0.23–0.82)	0.47 (0.24–0.85)	0.65 (0.44–0.95)			0.63 (0.32–1.16)		
*p* Value	0.196			<0.001	<0.001	<0.001	0.055			0.088		
**Education achievement**												
Under college	1.00	1.00	1.00	1.00	1.00	1.00	1.00	1.00	1.00	1.00	1.00	1.00
College or above	0.58 (0.42–0.81)	0.66 (0.46–0.95)	0.60 (0.42–0.85)	0.65 (0.47–0.91)	0.64 (0.44–0.94)	0.62 (0.43–0.90)	0.76 (0.58–0.99)	0.79 (0.58–1.06)	0.76 (0.57–1.01)	0.66 (0.45–0.98)	0.71 (0.46–1.11)	0.61 (0.41–0.92)
*p* Value	0.001	0.026	0.005	0.013	0.022	0.011	0.047	0.120	0.062	0.043	0.130	0.019
**Marital status**												
Single	1.00			1.00			1.00			1.00	1.00	1.00
Married	1.22 (0.86–1.71)			0.84 (0.58–1.19)			0.75 (0.56–1.00)			0.55 (0.33–0.86)	0.52 (0.31–0.84)	0.56 (0.34–0.89)
*p* Value	0.252			0.331			0.052			0.009	0.007	0.015
**Sleeping hours**												
<6	1.00			1.00			1.00			1.00	1.00	1.00
≥6	0.75 (0.49–1.17)			0.83 (0.55–1.31)			0.79 (0.56–1.13)			0.58 (0.36–0.97)	0.60 (0.37–1.02)	0.58 (0.36–0.98)
*p* Value	0.199			0.418			0.189			0.038	0.060	0.041
**W** **eekly working hours**												
<55	1.00	1.00	1.00	1.00	1.00	1.00	1.00	1.00	1.00	1.00		
≥55	2.20 (1.56–3.10)	2.00 (1.39–2.85)	2.08 (1.45–2.96)	1.71 (1.19–2.43)	1.71 (1.16–2.49)	1.71 (1.16–2.48)	1.93 (1.44–2.58)	1.85 (1.36–2.50)	1.85 (1.36–2.51)	1.38 (0.89–2.09)		
*p* Value	<0.001	<0.001	<0.001	0.004	0.007	0.007	<0.001	<0.001	<0.001	0.147		
**Hospital level**												
Secondary	1.00	1.00		1.00	1.00		1.00	1.00		1.00	1.00	
Tertiary	0.67 (0.49–0.93)	1.09 (0.69–1.70)		0.84 (0.62–1.16)	1.51 (0.95–2.40)		0.84 (0.65–1.08)	1.14 (0.79–1.64)		0.75 (0.52–1.09)	1.04 (0.62–1.75)	
*p* Value	0.016	0.719		0.304	0.079		0.176	0.481		0.127	0.875	

The 90th percentile as cut-off point, scores ≥95, 75, 75, and 75, respectively, determined personal burnout, client-related burnout, job dissatisfaction, and intention to leave. M1: Model 1, crude odds ratio. M2: Model 2, hospital level was included to adjust for ADPNR. M3: hospital level was not included because the standardized ADPNR was adjusted by the Taiwanese regulation.

**Table 3:** Results of multiple mediator model analysis.

Variable	Consequent											
	M1: Personal Burnout			M2: Client-Related Burnout			M3: Job Dissatisfaction			Y: Intention to Leave		
Antecedent	Coef.	s.e.	*p*	Coef.	s.e.	*p*	Coef.	s.e.	*p*	Coef.	s.e.	*p*
Standardized ADPNR	15.06	3.85	***	13.15	3.68	***	12.45	3.67	***	4.77	3.86	
**Mediators**												
M1: personal burnout	-	-	-	-	-	-	-	-	-	0.33	0.03	***
M2: client-related burnout	-	-	-	-	-	-	-	-	-	0.11	0.03	**
M3: job dissatisfaction	-	-	-	-	-	-	-	-	-	0.41	0.03	***
**Covariates**												
Ownership ^†^	2.59	1.14	*	1.73	1.09		2.61	1.09	*	2.75	1.14	*
Shift ^†^	1.11	0.45	*	1.13	0.43	**	0.65	0.43		−0.44	0.45	
Age ^§^	−0.73	0.85		−0.44	0.82		−0.56	0.81		−0.68	0.85	
Education achievement ^‖^	−4.84	1.20	***	−2.39	1.15	*	−3.35	1.15	**	1.34	1.21	
Marital status ^¶^	0.89	1.37		−0.96	1.31		−1.40	1.30		−3.55	1.37	**
Sleeping hours ^#^	−2.90	1.56		−0.61	1.49		−2.23	1.48		−1.98	1.56	
Weekly working hours ^Δ^	9.48	1.33	***	5.15	1.27	***	5.19	1.26	***	2.41	1.34	
*R-Square*	0.08 ***			0.04 ***			0.04 ***			0.35 ***		
Completely standardized effect on intention to leave	0.03 (0.01–0.05) ^ξ^			0.01 (0.00–0.02) ^ξ^			0.03 (0.01–0.05) ^ξ^			-		

Coef.: coefficient; s.e.: standard error. * *p* < 0.05, ** *p* < 0.01, *** *p* < 0.001. ^†^ ownership: “0” for public, “1” for private; ^‡^ shift: the major shift in the past three months, “0” for day shift, “1” for evening shift, “2” for night shift, “3” for rotating shift; ^§^ age: “0” under or equal to 25, “1” for 26 to 35, “2” for above or equal to 36; ^‖^ education achievement: “0” for junior college, “1” for college or above; ^¶^ marital status: “0” for single, “1” for married; ^#^ sleeping hours: sleeping hours per working day, “0” for less than 6 h, “1” for more than or equal to 6 h; ^Δ^ weekly working hours: set as the 75th percentile of the study population, “0” for less than 55 h, “1” for more than or equal to 55 h; ^ξ^ completely standardized effect (95% CI), estimated by bootstrap of 5,000 times.
